# Temporal Transcriptomic Analysis of Periodontal Disease Progression and Its Molecular Links to Systemic Diseases

**DOI:** 10.3390/ijms26051998

**Published:** 2025-02-25

**Authors:** Teerachate Nantakeeratipat, Chiharu Fujihara, Masahide Takedachi

**Affiliations:** 1 Department of Conservative Dentistry and Prosthodontics, Faculty of Dentistry, Srinakharinwirot University, Watthana, Bangkok 10110, Thailand; teerachate@g.swu.ac.th; 2 Department of Periodontology and Regenerative Dentistry, Osaka University Graduate School of Dentistry, Suita, Osaka 5650871, Japan; takedachi.masahide.dent@osaka-u.ac.jp

**Keywords:** periodontal disease, RNA sequencing, gene expression profiling, transcription factor enrichment analysis, transcriptomics, temporal analysis

## Abstract

Periodontal disease, a prevalent oral inflammatory condition, is implicated in exacerbating systemic diseases. However, the molecular mechanisms underlying this association remain unclear. In this study, we performed RNA sequencing of gingival tissue samples collected from a mouse model of periodontal disease at multiple time points to investigate dynamic transcriptomic changes during disease progression. Our analysis revealed distinct temporal gene expression patterns associated with the key inflammatory and immune response pathways. These findings suggest stepwise molecular progression in the periodontal inflammatory process, potentially contributing to systemic inflammation through shared signaling networks. We further identified specific genes and pathways that may mediate the bidirectional relationship between periodontal disease and systemic conditions such as cardiovascular disease and diabetes. By elucidating the temporal dynamics of molecular changes in periodontal disease, this study provides insights into the pathogenesis and its systemic implications. It identifies potential biomarkers and therapeutic targets for local and systemic disease management.

## 1. Introduction

Periodontitis, a chronic inflammatory condition of the periodontal tissue, is primarily initiated by the host immune response to periodontopathogens and their bacterial products [1]. The release of proinflammatory cytokines triggers a cascade of immune events intended to eliminate microbial insults but often results in collateral damage to the surrounding periodontal structures [2]. Although the pivotal role of inflammation in periodontitis has been studied extensively, our understanding of its complex pathogenesis remains limited. The disease progresses through multiple overlapping phases, each influenced by the dynamic interplay between microbes, hosts, and environmental factors. Previous studies have highlighted these complexities; however, they often fail to capture disease progression’s temporal and multifaceted nature [3].

Increasing evidence has linked periodontal disease to systemic conditions such as diabetes mellitus (DM), cardiovascular disease (CVD), metabolic disorders, rheumatoid arthritis (RA), and Alzheimer’s disease [4]. The molecular mechanisms underlying these associations involve the systemic dissemination of inflammatory mediators such as interleukin-6 (IL-6), tumor necrosis factor-alpha (TNF-α), and C-reactive protein (CRP), which can exacerbate systemic inflammation [5]. Additionally, periodontal pathogens, such as *Porphyromonas gingivalis (P. gingivalis*), can translocate into the bloodstream, contributing to the development of atheromatous plaques in cardiovascular diseases or altering insulin signaling pathways in DM [6]. For instance, *P. gingivalis* activates Toll-like receptor (TLR) pathways, leading to immune dysregulation and tissue damage [7]. In Alzheimer's disease, inflammatory mediators originating from periodontitis are hypothesized to enhance amyloid beta aggregation and neuroinflammation, further linking local periodontal inflammation to neurodegenerative processes [8]. These findings underscore the need for comprehensive studies to elucidate the molecular networks that link periodontal and systemic diseases. Despite substantial research efforts, the molecular mechanisms underlying these associations have not been fully elucidated, highlighting the need for further investigations to validate these links and explore the additional systemic implications of periodontitis.

Omics technologies, particularly transcriptomic analyses, have emerged as powerful tools for identifying risk factors, functional pathways, and molecular mechanisms associated with complex diseases [9]. In the context of periodontitis, these approaches provide an unbiased and comprehensive methodology for examining the multiphase nature of the disease. The application of omics technologies enables us to address gaps in our understanding of the dynamic processes underlying periodontitis pathogenesis and its broader implications for systemic health [10,11]. However, as the pathogenesis of periodontitis is time-dependent and multiphase, unraveling its molecular and cellular mechanisms remains a significant challenge. The lack of longitudinal studies and variability in disease manifestations further complicate efforts to derive definitive conclusions. Understanding temporal changes in gene expression and identifying key regulatory pathways during periodontitis progression is crucial for building a more comprehensive picture of disease progression. Furthermore, it is hypothesized that these regulatory pathways contribute to the exacerbation of systemic diseases by modulating shared inflammatory and immune response mechanisms.

This study aimed to advance the understanding of periodontitis by exploring its molecular dynamics and identifying the biological processes associated with its progression. Through transcriptomic analyses and time-course gene expression studies, we further seek to elucidate the molecular changes underlying periodontal disease progression and their potential systemic implications.

## 2. Results

### 2.1. Temporal Transcriptomic Data from Healthy and Periodontitis-Induced Gingival Tissues

**Figure 1:**
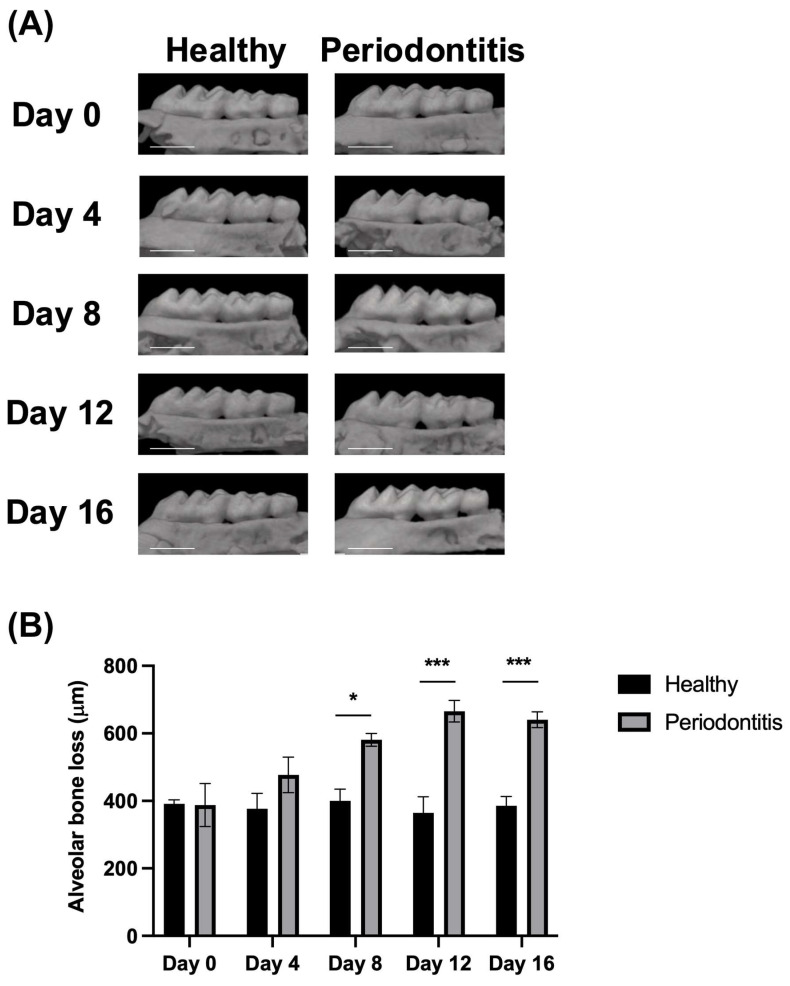
Validation of alveolar bone loss in the ligature-induced periodontitis mice. (**A**) The time-course micro-CT images of the maxillae in healthy and periodontitis groups. The representative micro-CT images among n = 3 at each time point and in each group are shown. Left: maxillae in the healthy group; right: maxillae in the periodontitis group. The scale bars: 1 mm. (**B**) The distance between the cementoenamel junction to the alveolar bone crest was calculated at the 4 roots of the first to third molars, and the sum was shown as alveolar bone loss. Black bars: healthy group, gray bars: periodontitis group. n = 3 in each time point and group. Data are present as mean ± standard error. * *p* < 0.05 and *** *p* < 0.001 vs. healthy group.

We used a mouse model of ligature-induced periodontitis to comprehensively elucidate its molecular pathogenesis. First, we assessed alveolar bone loss around the ligated teeth from days 0 to 16 using micro-computed tomography (micro-CT) imaging. Nonligated mice were used as controls (the healthy group). Micro-CT images revealed significant alveolar bone loss around the second molars in the ligated mice (the periodontitis group), particularly from day 8 to day 16, compared to the controls (Figure 1A,B). These findings confirmed the successful induction of periodontitis in the ligated mice. 

Using gingival tissues isolated from healthy and periodontitis-induced mice, RNA sequencing (RNA-seq) was performed to identify key genes and biological pathways involved in the pathogenesis of periodontitis. Temporal transcriptomic data from healthy and periodontitis-induced gingival tissues were analyzed to determine differential gene expression and associated pathways.

### 2.2. Differentially Expressed Genes (DEGs) Between Healthy and Periodontitis-Induced Gingival Tissues at Each Time Point

**Figure 2:**
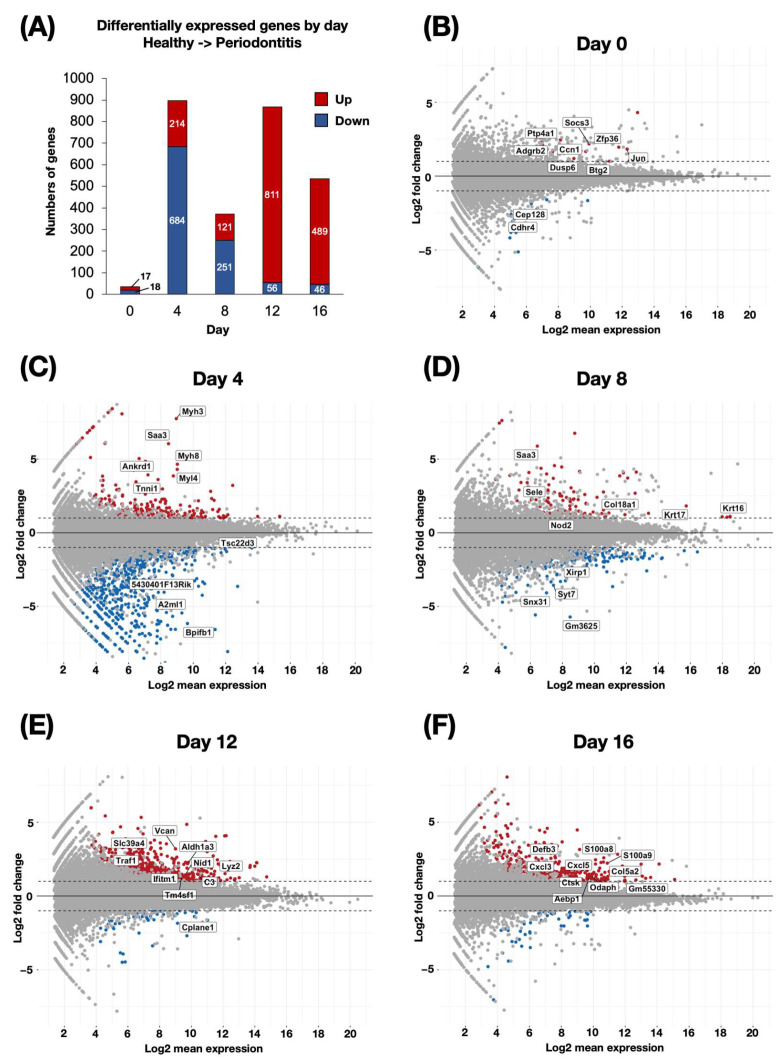
Temporal gene expression dynamics during periodontitis progression. (**A**) The number of differentially expressed genes (DEGs) at each time point (day 0, 4, 8, 12, and 16) comparing healthy and periodontitis-induced gingival tissues (n = 3 at each time point and in each group). Bars represent upregulated (red) and downregulated (blue) genes. (**B**–**F**) Volcano plots showing the distribution of DEGs at each time point (**B**) day 0, (**C**) day 4, (**D**) day 8, (**E**) day 12, and (**F**) day 16. Each dot represents a gene, with upregulated genes in red and downregulated genes in blue. Selected top genes based on fold-change and relevance are labeled. The *x*-axis represents the log2 mean expression and the *y*-axis represents the log2 fold change. The dashed horizontal lines indicate the threshold for significance.

We revealed transcriptional changes throughout disease development by comparing differential gene expression between periodontitis-induced and healthy gingival tissues at each time point. On day 0, 17 upregulated and 18 downregulated genes were identified. By day 4, upregulated genes increased to 214, whereas 684 were downregulated. On day 8, 121 upregulated and 251 downregulated genes were identified. A significant change was observed on day 12 with 811 upregulated and 56 downregulated genes. On day 16, upregulated genes decreased to 489, whereas 46 genes were downregulated (Figure 2A). These findings highlight the progressive transcriptional changes that occur in gingival tissue during the development of periodontitis.

**Figure 3:**
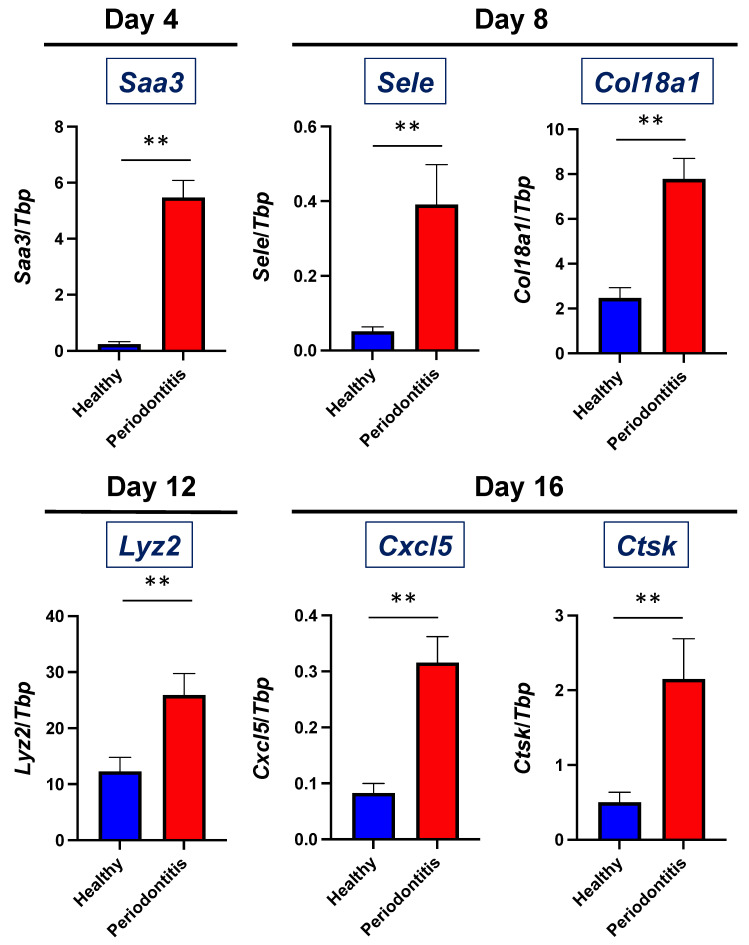
Validation of the upregulated gene expression in RNA-seq. The gene expression of *Saa3* (day 4), *Sele* (day 8), *Col18a1* (day 8), *Lyz2* (day 12), *Cxcl5* (day 16), and *Ctsk* (day 16) in the gingival tissues from healthy or periodontitis-induced mice was examined by qPCR. n = 5 on each day and in each group. Data are present as mean ± standard error. ** *p* < 0.01 vs. healthy.

We then analyzed the top 10 genes (|log2 fold change| > 1, *p*-adjusted value < 0.05) at each time point to identify the key genes underlying the development of periodontitis. On day 0, early responsive genes such as the suppressor of cytokine signaling 3 (*Socs3*) and Jun proto-oncogene (*Jun*) were upregulated. In addition, the upregulation of extracellular signal-regulated kinase (ERK) signaling-related genes, such as cellular communication network factor1 (*Ccn1*) and dual specificity phosphatase 6 (*Dusp6*), was detected in gingival tissues with periodontitis. Meanwhile, a significant downregulation was observed in genes such as cadherin-related family member 4 (*Cdhr4*) and centrosomal protein 128 (*Cep128*), which are associated with cellular adhesion and structural organization (Figure 2B). On day 4, a more pronounced transcriptional response was observed. Inflammatory genes, such as serum amyloid A3 (*Saa3*), and several genes involved in muscle contraction and the cytoskeleton, such as myosin heavy chain 3 (*Myh3*), myosin heavy chain 8 (*Myh8*), myosin light chain 4 (*Myl4*), ankyrin repeat domain 1 (*Ankrd1*), and troponin I1 (*Tnni1*), were significantly upregulated. Conversely, the downregulation of genes such as alpha-2-macroglobulin like 1 (*A2ml1*) and bactericidal/permeability-increasing protein (BPI)-fold containing family B member 1 (*Bpifb1*) was noted (Figure 2C). As the disease progressed to day 8, the upregulation of genes in the components of the epithelium and basement membrane, such as keratin 17 (*Krt17*), keratin 16 (*Krt16*), collagen type XVIII alpha 1 chain (*Col18a1*), and inflammatory and immune-related genes, such as nucleotide-binding oligomerization domain containing 2 (*Nod2*), selectin E (*Sele*), and *Saa3,* were detected. Concurrently, downregulated genes, including synaptotagmin 7 (*Syt7*) and sorting nexin 31 (*Snx31*), were linked to vesicular transport and cellular organization (Figure 2D). On day 12, genes related to the immune response, such as TNF receptor-associated factor 1 (*Traf1*), lysozyme 2 (*Lyz2*), complement component 3 (*C3*), and interferon-induced transmembrane protein 1 (*Ifitm1*), were prominently upregulated. The upregulation of genes encoding components of the extracellular matrix (ECM) and basement membrane, such as versican (*Vcan*) and nidogen-1 (*Nid1*), was also found in gingival tissue with periodontitis. Meanwhile, significant downregulation was observed in genes such as ciliogenesis and planar polarity effector complex subunit 1 (*Cplane1*), which are associated with the cell polarity required for cell migration (Figure 2E). Finally, by day 16, the upregulation of genes such as C-X-C motif chemokine ligand 5 (*Cxcl5*), C-X-C motif chemokine ligand 3 (*Cxcl3*), defensin beta 3 (*Defb3*), collagen type V alpha 2 chain (*Col5a2*), S100 calcium-binding protein A9 (*S100a9*), and S100 calcium-binding protein A8 (*S100a8*) demonstrated a convergence of sustained inflammatory signaling and connective tissue degradation (Figure 2F). To validate the RNA-seq results, we performed quantitative reverse transcription PCR (qPCR) analysis on a selection of genes that showed an upregulated expression. The qPCR results confirmed the RNA-seq data, demonstrating the consistent upregulation of these genes (Figure 3).

### 2.3. Gene Functions in Each Cluster During the Pathogenesis of Periodontitis

**Figure 4:**
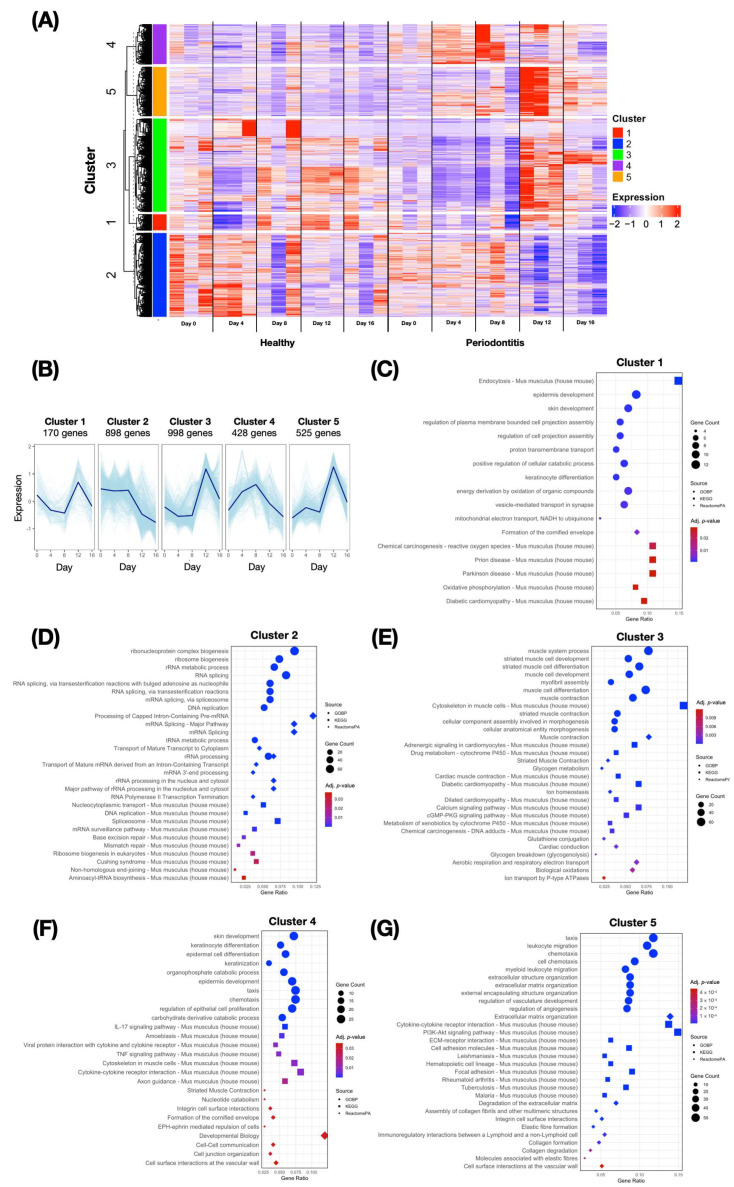
Gene expression profiles and functional enrichment analysis across five gene clusters in healthy and periodontitis-induced conditions. (**A**) Heatmap showing the temporal expression patterns of genes in clusters 1–5 during healthy and periodontitis-induced conditions. Gene expression is presented as a z-score, with red indicating upregulation and blue indicating downregulation. Clusters are color coded on the left. Time points are marked on the *x*-axis, with separate columns for healthy and periodontitis groups. (**B**) Dark blue lines representing the average gene expression pattern for each cluster over time (days 0–16) in periodontitis condition. Light blue lines represent the expression of all genes within each cluster. All expressions are presented as a z-score (**C**–**G**) of the functional enrichment analysis of the genes in clusters 1–5. Each symbol shows enriched Gene Ontology Biological Processes (GOBPs) (circle), Kyoto Encyclopedia of Genes and Genomes pathways (KEGGs) (square), or Reactome pathways (Reactome PA) (triangle) with the corresponding gene ratio (*x*-axis). The size of symbols represents the gene count in each term, and color indicates the adjusted *p*-value significance (red: high significance, blue: low significance).

The results of day-specific analysis alone cannot fully describe the progression of the disease. To gain deeper insight into the time-dependent pathophysiology of the disease, we conducted a time-course differential analysis. Candidate genes with an expression count greater than 10 and at least one time point showing significant up- or downregulation were selected for further investigation. Subsequently, we clustered the 3019 candidate genes into five distinct clusters based on their temporal expression patterns during periodontitis. Each cluster exhibited unique dynamic changes in gene expression, reflecting distinct biological processes associated with periodontitis progression and potential systemic implications (Figure 4A,B). 

Cluster 1, containing 170 genes, showed early downregulation during the onset of periodontitis, followed by a recovery from days 8 to 12. An enrichment analysis revealed pathways related to endocytosis, epidermal development, and skin development, as well as reactive-oxygen-species-related chemical carcinogenesis, energy-metabolism-related oxidative phosphorylation, and mitochondrial electron transport. In addition to biological pathways, the analysis revealed relationships with systemic diseases, such as prion disease, Parkinson’s disease, and diabetic cardiomyopathy (Figure 4C).

Cluster 2, comprising 898 genes, showed sustained downregulation throughout the time. This cluster was enriched in pathways mostly related to cellular processes, including ribonucleoprotein complex biogenesis, ribosome biogenesis, and RNA splicing. The large number of enriched pathways in cluster 2 underscores the impact of inflammation on essential cellular processes. Disruptions in RNA and DNA functions are critical for downstream protein expression. Additionally, systemic conditions such as Cushing syndrome were shown in the analysis (Figure 4D).

Cluster 3, with 998 genes, displayed a slight downregulation from day 0 to day 8, followed by an upregulation that peaked on day 12. Enriched pathways included muscle-related pathways such as muscle system processes and striated muscle cell development, metabolism-related pathways such as drug metabolism-cytochrome p450 and glycogen metabolism, and signaling pathways such as the calcium and cyclic guanosine monophosphate-protein kinase G (cGMP-PKG) signaling pathways. This analysis revealed several cardiovascular-system-associated pathways, including cardiac muscle contraction, diabetic cardiomyopathy, and cardiac conduction (Figure 4E).

Cluster 4 included 428 genes that were upregulated in the early stages of periodontitis but downregulated in the later stages. An enrichment analysis revealed pathways such as epidermal cell differentiation, keratinocyte differentiation, and cytokine-mediated signaling, including Interleukin-17 (IL-17) signaling and cytokine–cytokine receptor interactions (Figure 4F).

Cluster 5 consisted of 525 genes with a gradual increase in expression over time, which peaked at the later stages of periodontitis. The enriched pathways included leukocyte migration, chemotaxis, cytokine–cytokine receptor interactions, phosphatidylinositol 3-kinase-protein kinase B (PI3K-Akt) signaling, and extracellular-related pathways such as extracellular structure organization, ECM organization, ECM-receptor organization, degradation of ECM, and vascular-related pathways. Systemic conditions were identified, including RA, tuberculosis, and leishmaniasis (Figure 4G). The increase in immune-related pathways in cluster 5 highlights the inflammatory characteristics of periodontitis and their association with systemic diseases.

### 2.4. Transcription Factor (TF) Enrichment Across Gene Clusters

**Figure 5:**
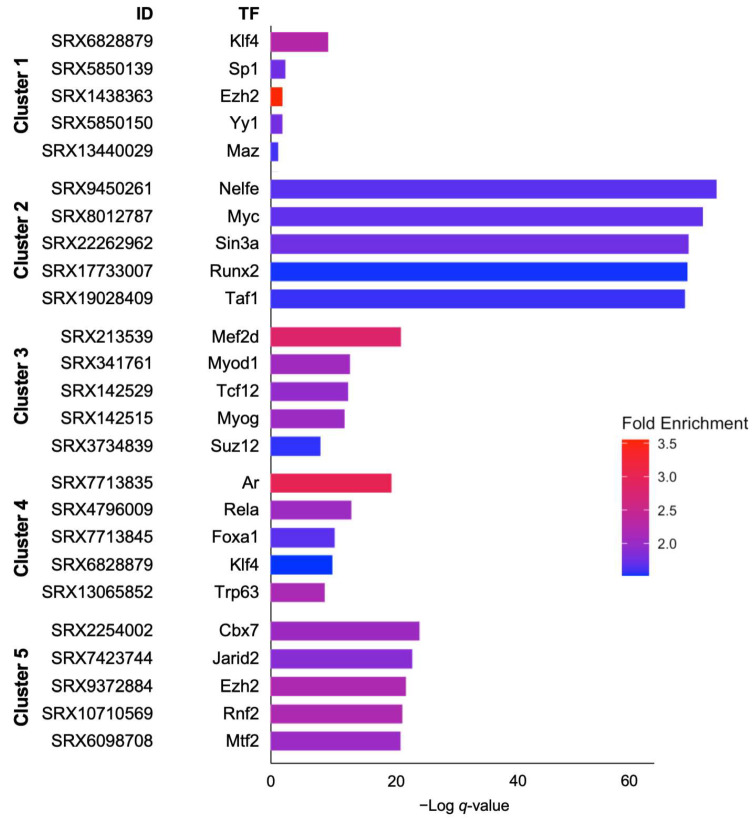
Enrichment of transcription factors (TFs) across five distinct clusters of experimental data. The graph presents TFs potentially regulating gene expression in each of the 5 clusters (cluster 1 to cluster 5) derived from RNA-seq analysis. The *x*-axis represents the negative log-transformed *q*-values (−Log *q*-value), which indicates the statistical significance of TF enrichment. Higher values correspond to greater significance. The *y*-axis lists the TFs associated with specific experimental IDs (reference datasets from Sequence Read Archive (SRA) data) within each cluster. Bar colors correspond to fold enrichment, with a gradient from blue to red. Blue: lower enrichment; red: higher enrichment.

To further investigate the regulatory mechanisms underlying the DEGs in each cluster, we conducted an enrichment analysis using ChIP-Atlas, focusing on TF-binding sites and epigenetic markers associated with the upregulated genes. The distinct TF enrichment patterns across each cluster are shown (Figure 5).

Genes from cluster 1 were enriched in TFs, such as krüppel-like factor 4 (*Klf4*), specificity protein 1 (*Sp1*), enhancer of zeste homolog 2 (*Ezh2*), yin yang 1 (*Yy1*), and myc-associated zinc finger protein (*Maz*). These TFs have been implicated in transcriptional activation and chromatin remodeling. *Klf4* and *Sp1* are key mediators of transcriptional responses to inflammatory stimuli, whereas *Ezh2* and *Yy1* function as epigenetic regulators that influence chromatin accessibility and gene expression during early inflammatory responses. 

Genes from cluster 2 were enriched for TFs such as negative elongation factor E (*Nelfe*), myelocytomatosis oncogene (*Myc*), switch-independent 3a (*Sin3a*), and runt-related transcription factor 2 (*Runx2*). *Nelfe* and *Myc* are associated with transcriptional elongation and cell proliferation, respectively, while *Sin3a* is involved in chromatin regulation. *Runx2*, a critical regulator of osteogenic gene expression, reflects transcriptional activity linked to bone metabolism and remodeling. 

Genes from cluster 3 included TFs such as myocyte enhancer factor 2D (*Mef2d*), myogenic differentiation 1 (*Myod1*), transcription factor 12 (*Tcf12*), myogenin (*Myog*), and polycomb protein Suz12 (*Suz12*). These factors are pivotal for mesenchymal differentiation and tissue remodeling. *Mef2d* activates transcription in the muscle and mesenchymal systems, *Myod1* and *Tcf12* regulate cellular differentiation, *Myog* directs muscle development and repair, and *Suz12* regulates chromatin remodeling and gene silencing.

Genes from cluster 4 were enriched for androgen receptor (*Ar*), p65 (*Rela*), forkhead box A1 (*Foxa1*), *Klf4*, and tumor protein p63 (*Trp63*). *Ar* is a nuclear receptor involved in androgen signaling and hormonal regulation, whereas *Rela* is a subunit of the nuclear factor kappa B (NF-κB) complex, which orchestrates inflammatory and immune responses by regulating proinflammatory cytokine expression. *Foxa1* functions as a pioneering factor facilitating chromatin accessibility. *Trp63* is critical for epithelial development and stem cell maintenance.

Genes from cluster 5 included TFs such as chromobox protein homolog 7 (*Cbx7*), Jumonji, an AT-rich interaction domain containing 2 (*Jarid2*), *Ezh2*, ring finger protein 2 (*Rnf2*), and metal response element-binding transcription factor 2 (*Mtf2*). These factors play key roles in chromatin remodeling and transcriptional repression. *Cbx7*, *Jarid2*, and *Ezh2* are core polycomb complex components, *Rnf2* supports ubiquitination, and *Mtf2* aids polycomb recruitment to target genes.

## 3. Discussion

The findings of this study significantly enhanced our understanding of the molecular mechanisms underlying periodontitis and its systemic implications. We illustrated stage-specific biological processes by integrating transcriptomic data with pathway enrichment and TF analysis. Furthermore, TFs interact with specific regulatory sequences in DNA within the cell nucleus by promoting or inhibiting gene transcription. As key regulators of gene expression, TFs play a critical role in orchestrating biological processes, and their dysfunction is closely linked to various human diseases [12,13]. Identifying TFs in this study bridges local periodontal pathology with systemic diseases. Previous studies identified certain genes and master regulators in experimental periodontitis models in a time-point-specific manner [14]. Our analysis identified some of these previously reported molecules while also uncovering novel molecules through temporal analysis. These findings advance our molecular understanding of periodontitis and highlight practical biomarkers and therapeutic targets, offering a foundation for precision medicine approaches that address both local periodontal damage and broader systemic health challenges.

In this study, we selected the time points based on the progression of periodontal disease observed in our ligature-induced periodontitis model using 8-0 size silk ligature. Our micro-CT analysis revealed a gradual increase in alveolar bone loss from day 0 to day 12, indicating continuous disease progression (Figure 1B). However, between days 12 and 16, alveolar bone loss plateaued, suggesting a transition from the active destruction phase to a more resting phase. At the histological levels, early inflammatory responses occurred within the first 4 days. Peak tissue destruction and osteoclast appearance were observed between days 8 and 12. Late-stage resolution mechanisms began to appear by day 16. These observations are correlated with the RNA-seq results, such as the upregulation of immune-related genes on day 4, the highest levels of inflammatory and matrix degradation pathways on days 8 and 12, and tissue repair and immune modulation on day 16. The observations provide a comprehensive framework for understanding the dynamic transcriptomic changes occurring throughout periodontal disease progression. Thus, the selected time points effectively capture key molecular and structural changes, allowing for a detailed characterization of the disease trajectory.

Multiple biological and environmental factors can influence gene expression in mouse models. In our study, we controlled for major confounders by using age-matched and genetically homogeneous C57BL/6J mice. All mice were housed under identical conditions (temperature, light cycle, and diet). The same genes and pathways identified in our study have been reported in previous studies using the ligature-induced mice periodontitis model [11,15,16], reinforcing the reproducibility and biological validity of our transcriptomic analysis. Additionally, it is well known that the oral microbiota composition differs between mice and humans [17], which could influence the periodontal disease phenotype. However, despite these species-specific differences, our analysis identified pathways that are also implicated in human periodontal disease progression [18,19,20], supporting the relevance of our findings. Our clustering-based approach mitigates individual variability by focusing on consistent temporal expression patterns rather than isolated gene fluctuation. 

### 3.1. Dynamic Gene Expression in Periodontitis Progression

The temporal analysis revealed significant gene expression changes that aligned with the distinct stages of periodontitis. The early upregulation of *Socs3* and *Jun* reflects tissue immune response and stress adaptation. *Socs3* is critical in modulating cytokine signaling by acting as a negative regulator to prevent excessive inflammation. In contrast, *Jun* drives gene expression in cell proliferation and stress responses [21,22]. These mechanisms are consistent with acute immune activation observed in early periodontitis, where gingival tissues attempt to manage microbial invasion and inflammatory signaling [2]. The downregulation of genes such as *Cdhr4* during the early stages highlights compromised epithelial integrity. The loss of the epithelial barrier function is a hallmark of gingival tissue changes in periodontitis, which creates a vulnerable environment for microbial biofilm expansion [23]. On day 4, the upregulation of *Myh3*, *Myh8*, and *Myl4* suggested active tissue remodeling involving contractile proteins, reflecting a healing response to inflammation. 

In contrast, the downregulation of *Bpifb1*, an antimicrobial gene, indicates weakened bacterial defense, potentially exacerbating the microbial load and inflammation [24]. During the mid-phase (day 8), the upregulation of *Nod2*, a key pattern recognition receptor, and *Sele*, which mediates leukocyte adhesion, reflects ongoing immune surveillance and sustained inflammatory signaling. These genes were aligned with persistent immune cell infiltration observed in chronic periodontitis [25]. *Nod2* is critical for recognizing bacterial components, amplifying inflammatory responses, and driving NF-κB activation, a central pathway in periodontitis [26]. Similarly, *Sele* facilitates neutrophil adhesion and transmigration, contributing to gingival swelling and tissue stress [27]. On day 12, upregulated genes reflected active inflammatory and reparative processes. These genes are involved in ECM remodeling (*Vcan* and *Nid1*), immune responses (*Lyz2*, *C3*, and *Traf1*), and cellular defense mechanisms (*Ifitm1*, *Aldh1a3*, and *Slc39a4*), indicating an attempt to counteract tissue damage while sustaining inflammation. Conversely, the downregulation of *Cplane*1, a gene involved in ciliogenesis, may imply a reduced capacity for cellular organization and tissue homeostasis, potentially affecting proper tissue repair during the progression [28]. This dual dynamic underscores the heightened reparative yet unresolved inflammatory state. In the late stages, *S100a8* and *S100a9*, which encode damage-associated molecular patterns, and *Cxcl5* and *Cxcl3*, which encode chemokines, are prominently upregulated, highlighting the persistent recruitment of neutrophils, a hallmark of periodontitis [29]. Their role emphasizes a chronic inflammatory state in patients with advanced periodontitis.

### 3.2. Cluster-Specific Insights into Periodontitis Pathogenesis

The cluster analysis identified five distinct gene expression patterns during periodontitis progression, each associated with specific biological pathways and functions. Additionally, an enrichment analysis of the TFs in each cluster illustrated their potential regulatory mechanisms. These clusters provide insight into the molecular drivers of the disease while reflecting biological processes that may influence its nature.

Cluster 1 was enriched in oxidative phosphorylation, endocytosis, and epidermal development pathways. These findings suggest that the initial mitochondrial dysfunction and a compromised epithelial barrier are critical for maintaining gingival integrity. Impaired oxidative phosphorylation (OXPHOS) may lead to an inadequate energy supply, contributing to tissue dysfunction during the early stages of periodontitis [30]. This is supported by the finding that the inhibition of lysosomal acid lipase (LAL) activity, which plays a crucial role in maintaining bioenergetic processes by driving OXPHOS, in periodontal ligament cells significantly reduces adenosine triphosphate (ATP) production and OXPHOS capacity, emphasizing its importance for cellular energy metabolism and periodontal tissue homeostasis [31]. The recovery phase reflects the cellular efforts to restore energy homeostasis and epithelial repair, potentially mitigating further tissue damage. The prominent role of *Klf4* underscores the importance of transcriptional regulation in orchestrating the dynamic interplay among inflammation, tissue damage, and repair during periodontitis progression [32]. Additionally, other enriched TFs regulate inflammation and tissue remodeling, underscoring the complex interplay between the repair and inflammation characteristics of periodontitis.

Cluster 2 was enriched in pathways related to ribosome biogenesis, RNA splicing, and ribonucleoprotein complex assembly, suggesting the long-term suppression of protein synthesis and cellular function under chronic inflammation [33]. TFs such as *Myc* and *Nelfe* govern cell proliferation and transcription elongation [34]. Their downregulation reflects impaired tissue regeneration in chronic inflammatory conditions such as periodontitis. *Runx2*, a critical TF for osteoblast differentiation and bone formation, likely contributes to this cluster’s impaired regenerative processes of periodontal tissue destroyed by periodontitis [35]. Other chromatin regulators, such as *Taf1* and *Sin3a*, which are also responsible for the transcriptional dysregulation of tissue development, were observed in this cluster [36]. Collectively, these factors highlight the effects of chronic inflammation and transcriptional repression on tissue regeneration.

Cluster 3 was enriched in ECM organization, glycogen metabolism, and muscle system processes. Glycogen metabolism pathway enrichment suggests increased energy demand to sustain tissue repair during periodontitis [37]. *Mef2d*, involved in mitochondrial biogenesis and energy metabolism, may mediate processes critical for tissue remodeling [38]. *Myod1*, traditionally associated with muscle repair, may support mesenchymal stem cell differentiation in periodontal tissues [39]. *Tcf12* facilitates ECM organization and fibroblast activity and promotes tissue repair. *Myog* presumably contributes to connective tissue stabilization under inflammatory stress [40]. *Suz12*, an epigenetic regulator, may favor reparative pathways by silencing inflammatory genes, reflecting a dynamic balance between inflammation and the resolution of periodontitis [41].

The dynamics observed in cluster 4, associated with immune activation, highlighted pathways such as IL-17 signaling and cytokine–cytokine receptor interactions, which are pivotal in the pathogenesis of periodontitis. Regulated by the transcription factor *Rela*, this cluster underscores the importance of NF-κB activation in producing proinflammatory cytokines in periodontal tissue [42]. TFs such as *Foxa1* and *Trp63* significantly regulate epithelial integrity and immune responses [43,44]. Their downregulation may lead to epithelial dysfunction, increased susceptibility to chronic inflammation, and delayed healing.

Cluster 5 reflected the persistent inflammatory state of advanced periodontitis, as evidenced by pathways such as leukocyte migration, PI3K-Akt signaling, and ECM degradation. The TFs such as *Cbx7*, *Jarid2*, *Rnf2*, and *Mtf2* are associated with chromatin remodeling and are involved in these signaling pathways [45]. For example, *Cbx7* facilitates gene silencing by recognizing histone modifications and compacting chromatin [46]. Periodontitis may repress the genes involved in tissue repair, sustain inflammatory pathways, and limit regeneration. *Jarid2* regulates inflammatory gene expression, suppresses tissue remodeling pathways, and perpetuates chronic inflammation [47]. Collectively, these transcription factors highlight the critical role of epigenetic regulation in sustaining chronic inflammation, inhibiting tissue repair, and promoting ECM degradation.

### 3.3. Implications in Systemic Conditions

#### 3.3.1. Diabetes Mellitus (DM) and Metabolic Disorders

Pathways, such as OXPHOS and mitochondrial electron transport, highlight mitochondrial dysfunction, a key feature linking periodontitis to systemic insulin resistance and impaired glucose metabolism. Mitochondrial dysfunction promotes the accumulation of reactive oxygen species, interfering with insulin signaling and amplifying systemic metabolic stress [48]. Glycogen metabolism and calcium signaling reflect the energy demand for tissue repair in periodontitis. The dysregulation of glycogen metabolism in periodontal tissue may induce a systemic energy imbalance, particularly in individuals with DM [49]. It may also affect calcium signaling, critical for insulin secretion and glucose uptake. This further connects periodontal healing processes to systemic metabolic disruptions [50]. TFs, such as *Mef2d,* which regulates mitochondrial biogenesis, and *Suz12,* which is involved in inflammatory gene regulation, provide molecular links to systemic glucose dysregulation and insulin resistance. In addition, a recent study reported that *Rela*, a subunit of the NF-κB complex, is upregulated in macrophages of periodontitis patients with type 2 diabetes [51]. The systemic inflammatory burden of periodontitis may extend to other metabolic disorders such as obesity and metabolic syndrome. Pathways such as PI3K-Akt signaling are pivotal for tissue repair in periodontitis and systemic metabolic regulation [52]. Interestingly, a recent report suggested that epigenetic modification-related molecules, predominantly observed in cluster 5, may be critical links between immune function and metabolism [53]. Therefore, the dysregulation of these pathways mirrors chronic low-grade inflammation and metabolic imbalance. 

#### 3.3.2. Cardiovascular Diseases (CVDs)

The connection between periodontitis and CVD is driven by shared inflammatory pathways, oxidative stress, and vascular dysfunction [54]. Proinflammatory cytokines and bacterial components, such as lipopolysaccharides (LPS), released during periodontal inflammation contribute to endothelial dysfunction and vascular inflammation, key factors in atherosclerosis and myocardial dysfunction. Pathways such as cardiac muscle contraction and calcium signaling highlight the systemic effects of periodontitis on cardiac function, including its potential impact on arrhythmias and myocardial contractility. PI3K-Akt signaling and ECM degradation mirror the processes of vascular remodeling and plaque instability, which are central to atherosclerosis progression [55]. Transcription factors such as *Rela* and *Mef2d* emphasize the molecular link between periodontitis and CVD. For example, the dysregulation of *Mef2d* has been implicated in conditions such as dilated cardiomyopathy and cardiac hypertrophy, reflecting shared inflammatory and metabolic axes [56,57]. Clinically, periodontitis is associated with increased levels of systemic markers, such as CRP and increased carotid intima-media thickness, indicative of atherosclerosis [58].

#### 3.3.3. Rheumatoid Arthritis (RA)

Periodontitis and RA share similar pathophysiological mechanisms characterized by chronic inflammation, immune dysregulation, and connective tissue destruction [59]. The pathways, such as IL-17 signaling and cytokine–cytokine receptor interactions, underline the important role of proinflammatory cytokines like IL-17, TNF-α, and IL-6 in driving neutrophil recruitment and amplifying the inflammatory response during periodontitis. These cytokines contribute to synovial inflammation and joint damage in RA, paralleling their role in periodontal tissue destruction [60]. The persistence of inflammatory signaling, including pathways such as leukocyte migration and ECM degradation, reflects the chronic immune activation observed in both conditions. TFs such as *Foxa1* and *Trp63*, implicated in epithelial barrier integrity and immune regulation, also play critical roles in both diseases. *Jarid2* and *Mtf2*, involved in chromatin remodeling and gene repression, contribute to the persistent inflammatory environment and tissue destruction observed in the advanced stages of RA and periodontitis.

#### 3.3.4. Neurodegenerative Diseases

Emerging evidence links periodontitis to neurodegenerative diseases, such as Alzheimer’s disease, Parkinson’s disease, and other forms of cognitive defects. The systemic inflammation, oxidative stress, and microbial dissemination associated with periodontitis contribute to mechanisms implicated in neurodegeneration [61]. The insights from this study highlight the molecular pathways and transcription factors that offer new perspectives on this connection. Pathways such as OXPHOS and mitochondrial electron transport indicate that mitochondrial dysfunction and oxidative stress are critical links between periodontitis and neurodegenerative diseases. Interestingly, the disruption of metabolism-related *Mef2d* was observed in mice and patients with Parkinson’s disease [38]. IL-17 signaling and cytokine–cytokine receptor interactions highlight the role of systemic immune activation in neuroinflammation. Proinflammatory cytokines, including IL-17, which increase during periodontitis, can cross the blood–brain barrier and activate microglial cells, thereby promoting the release of neurotoxic factors [62,63]. This chronic activation of microglia contributes to amyloid beta deposition and tau hyperphosphorylation, pathological features of Alzheimer’s disease [64]. TFs, such as *Ezh2* and *Suz12*, activated in periodontitis, provide additional mechanistic insights. *Ezh2* plays a role in regulating neuroinflammation. Interestingly, the dysregulation of *Ezh2* in the brain can increase proinflammatory signaling, exacerbating neuronal damage [65,66]. Similarly, *Suz12* may influence the balance between neuronal repair and inflammation, linking periodontitis with neurodegenerative diseases [67].

### 3.4. Potential Biomarkers and Therapeutic Targets for Disease Management

This study highlights the key biomarkers and therapeutic targets that can be used to treat periodontitis and its systemic effects. Biomarkers associated with mitochondrial dysfunction and oxidative stress are among the most interesting discoveries. These genes indicate early energy deficits in the periodontal tissue and play an essential role in tissue repair and immune responses. Generally, during the disruption of mitochondrial function, cells produce less ATP, resulting in decreased energy-dependent processes and an increased inflammatory response [68]. This induces the destruction of the periodontal tissue. Similar mitochondrial defects are also observed in systemic conditions, such as DM, CVD, and neurodegenerative diseases, emphasizing shared metabolic vulnerabilities [69]. 

Additionally, inflammatory biomarkers such as IL-17, TNF-α, and IL-6 highlight direct links between local periodontal disease and systemic inflammation. Enzymes breaking down the ECM, such as matrix metalloproteinases, reflect advanced periodontal damage and play roles in systemic diseases such as RA and cancer [70,71]. These biomarkers are valuable tools for detecting disease progression and systemic risk. This study also highlighted several therapeutic targets that may provide new possibilities for interventions in treating periodontitis and systemic diseases. One of the interesting molecules is *Klf4*. It plays a crucial role in maintaining the integrity of the epithelial barrier and regulating anti-inflammatory responses [72]. By promoting keratinocyte differentiation and mitigating oxidative stress, *Klf4* may aid in preserving gingival health during inflammatory challenges in periodontitis. Enhancing Klf4 protein activity may help restore tissue homeostasis and protect against systemic inflammatory complications such as CVD. 

Currently, Klf4 has been proposed as a biomarker for several systemic conditions, such as atherosclerosis and lung cancer [73,74]. As the pathogenesis of periodontitis is indicated by its multiple stages, it is interesting that monitoring Klf4 expression could provide insights into the severity and stage of atherosclerosis. While high Klf4 in endothelial cells and macrophages is protective and may reflect early or stable disease stages, reduced Klf4 is pro-atherosclerotic, indicating increased inflammation, foam cell formation, and plaque instability [74]. Similarly, *Mef2d* regulates energy metabolism during tissue repair and is essential for mitochondrial biogenesis and cellular energy homeostasis [75]. Its role in coordinating tissue remodeling underscores its potential as a therapeutic target. Targeting *Mef2d* may alleviate energy deficits in periodontal tissues and address metabolic disruptions commonly observed in systemic disorders such as DM and CVD. 

Interestingly, this study reports that *Mef2d* plays a crucial role as a key mediator linking LPS-stimulated inflammation and lung cancer progression by influencing the cancer microenvironment and cellular behavior. Therefore, it may serve as a potential biomarker for the transition from chronic inflammation to lung cancer [76]. Given that periodontitis is characterized by chronic inflammation and bacterial infection, it is plausible that *Mef2d* may play a similar role in its pathogenesis. The key signaling pathways involved in the progression of periodontitis may also serve as therapeutic targets. For instance, PI3K-Akt and IL-17 signaling are crucial for managing inflammation and tissue remodeling. The modulation of these pathways could help control periodontal damage and systemic inflammatory diseases. 

Furthermore, transcription factors, such as *Cbx7* and *Jarid2*, which regulate chromatin structure, offer additional avenues to epigenetically manage inflammation and tissue repair. As epigenetic regulation becomes a more interesting strategy, TFs, such as *Ezh2*, *Suz12*, and *Jarid2*, contribute to the resolution of inflammation resolution and tissue repair by modulating gene activity through epigenetic mechanisms. These factors can silence proinflammatory genes via histone methylation, reducing excessive immune responses and preventing chronic inflammation [77]. Simultaneously, they promote tissue regeneration by regulating genes involved in ECM remodeling, fibroblast activity, and stem cell differentiation [78]. Targeting these TFs offers promising therapeutic strategies for restoring the balance between inflammation and tissue healing in periodontitis and the systemic conditions associated with periodontitis. 

### 3.5. Limitations and Future Directions

This study identified key genes and pathways associated with periodontal disease progression and discussed their potential roles in exacerbating systemic diseases based on early reports. However, whether these shared genes and pathways directly contribute to the deterioration of systemic diseases when upregulated in periodontal disease remains to be experimentally validated. Future studies incorporating in vivo animal models will be necessary to determine the causal relationship between periodontitis-induced gene expression changes and systemic disease progression. Additionally, we propose investigations using a combination of mathematical modeling and experimental approaches. We previously developed a mathematical model to describe the progression of periodontitis driven by oral bacteria [79]. A similar approach can be applied in the proposed study. For example, by analyzing gene expression profiles and phenotypic changes in periodontitis with DM and CVD using clinical or experimental data and applying them to the mathematical model, we can uncover the pathways mediating the connection between periodontitis and systemic diseases. This integrated approach not only enhances our understanding of the shared mechanisms but also potentially enables more personalized periodontitis treatments tailored to an individual’s systemic health status. 

Our findings highlight the potential of therapies that address both periodontal and systemic health and advance the concept of precision medicine. By identifying key TFs, pathways, and metabolic disruptions, this study opens new avenues for future research. Further investigation of the identified molecules will deepen our understanding of their functions in both periodontal and systemic contexts, paving the way for novel therapeutic interventions. 

## 4. Materials and Methods

### 4.1. Mice

C57BL/6J mice were obtained from Japan SLC (Shizuoka, Japan). The animals were housed under controlled conditions at Osaka University, adhering strictly to institutional guidelines. They were kept in a temperature-regulated environment with a 12-hour light/dark cycle and had unrestricted access to food and water. The mice were fed a standard chow diet (MF Oriental Yeast Co., Ltd., Tokyo, Japan). Prior to performing any experimental procedures, approval was obtained from the Institutional Animal Care and Use Committee of the Osaka University Graduate School of Dentistry (R02-008). The study protocol ensured the ethical treatment of animals and complied with the ARRIVE 2.0 guidelines for animal research.

### 4.2. Developing a Mouse Ligature-Induced Periodontitis Model

We induced periodontitis in mice (the periodontitis group) as previously described with some modification [80]. Briefly, an 8–0 silk ligature (CROWNJUN, KONO Seisakusho Co., Ltd., Chiba, Japan) was carefully tied around the maxillary second molars of six- to eight-week-old male and female mice under general anesthesia. Anesthesia was achieved using an intraperitoneal injection comprising 0.3 mg/kg medetomidine, 4.0 mg/kg midazolam, and 5.0 mg/kg butorphanol. The ligature was gently secured to minimize tissue damage. For the control group (the healthy group), the maxillary second molars were unligated. Mice were randomly divided into experimental groups after a one-week acclimatization period, with group assignments performed by an independent individual. Animals were euthanized on days 0, 4, 8, 12, and 16 following disease induction using carbon dioxide, ensuring humane endpoints. Prior to euthanasia, the ligature’s presence around the teeth in the periodontitis group was confirmed. Mice from which the ligature was lost were excluded from subsequent analyses.

### 4.3. Quantitative Assessment of Alveolar Bone Loss

Maxillae were collected and subjected to imaging using the R_mCT2 3D X-ray micro-computed tomography system (Rigaku, Tokyo, Japan). The obtained images were processed into 2D images with the TRI/3D-BON software version R.11.00.00.2-H-64 (RATOC System Engineering, Tokyo, Japan). Alveolar bone loss was quantified by measuring the cumulative distances from the cementoenamel junction to the alveolar bone crest on the buccal surface at four specific points: (1) the distal root of the first maxillary molar; (2) the mesial root of the second maxillary molar; (3) the distal root of the second maxillary molar; and (4) the mesiobuccal root of the third maxillary molar. Measurements were carried out using ImageJ software (version 1.0) (National Institutes of Health, Bethesda, MD, USA). 

### 4.4. RNA-seq Analysis

The gingival tissues around the 2nd molars of maxillae were collected, and total RNA was extracted using the TRIzol Reagent (Thermo Fisher Scientific, Waltham, MA, USA) in combination with Phasemaker Tubes (Thermo Fisher Scientific). RNA quality was evaluated with a 2100 Bioanalyzer (Agilent, Santa Clara, CA, USA) to ensure suitability for downstream applications. Poly(A)+ RNA was enriched utilizing Sera-Mag Oligo(dT)-Coated Magnetic Particles (GE Healthcare Life Sciences, Marlborough, MA, USA). Subsequently, cDNA synthesis and strand-specific library preparation were conducted using the dUTP protocol as previously described [81]. Libraries were sequenced on an Illumina NextSeq 500 platform (San Diego, CA, USA), generating 75 bp single-end reads. Adaptor sequences were removed, and quality control was performed using Trim Galore (version 0.6.6., https://github.com/FelixKrueger/TrimGalore (accessed on 5 December 2024)). 

Reads were aligned to the GRCm39 mouse reference genome using the STAR aligner version 2.6.1c (accessed on 5 December 2024) [82], and unique read counts were obtained using featureCounts version 2.0.3 (accessed on 5 December 2024) [83]. A differential gene expression analysis was carried out with the DESeq2 package [84], comparing the gingival tissues from mice in the healthy and periodontitis groups at each time point and for the time-course analysis. The genes meeting the criteria of an adjusted *p*-value < 0.05 and expression count greater than 10 were selected for further analyses. Hierarchical clustering was performed using the hclust package to identify patterns across five time points. Enrichment analyses for biological pathways were conducted using the Kyoto Encyclopedia of Genes and Genomes (KEGGs) [85], Gene Ontology Biological Processes (GOBPs), and Reactome pathway (Reactome PA) database [86] with the clusterProfiler package [87]. The analyses and visualizations of the RNA-seq data were performed with R version 4.4.2 (R Foundation for Statistical Computing, Vienna, Austria) in R Studio version 2024.09.1+394 (accessed on 5 December 2024) (Posit PBC, Boston, MA, USA).

### 4.5. Quantitative Reverse Transcription PCR (qPCR)

Total RNA was isolated from the gingival tissues using the TRIzol Reagent and Phasemaker Tubes (Thermo Fisher Scientific). cDNA was generated using the High-Capacity RNA-to-cDNA Kit (Thermo Fisher Scientific), and qPCR was performed using the StepOnePlus Real-time PCR System with Power SYBR Green PCR Master Mix and specific PCR primers (Thermo Fisher Scientific). The primer sequences used for qPCR are listed in Appendix A. TATA-box binding protein (*Tbp*) was used as the internal control. The results were analyzed using the ΔCt method. The relative gene expression to *Tbp* was calculated.

### 4.6. Transcription Factor (TF) Enrichment Analysis

A TF enrichment analysis was performed using the ChIP-Atlas 3.0, an integrative database of publicly available Chromatin Immunoprecipitation sequencing (ChIP-seq) data, via a web-based platform (https://chip-atlas.org/) (accessed on 22 December 2024) [88]. The genes from each cluster, identified through an RNA-seq analysis, were used as input for the ChIP-Atlas enrichment analysis. The analysis was conducted under selected options (experiment type: ChIP: TFs and others, cell type class: all cell types, threshold for significance: 50). The enrichments were calculated by comparing the overlap between the input gene list and ChIP-seq peaks from the database.

### 4.7. Statistical Analyses

The statistical analyses were performed using GraphPad Prism10 or R Studio version 4.4.2. A statistical comparison of a two-way analysis of variance for multivariate analysis was performed in Figure 1B. For comparisons between the two groups in Figure 3, the Mann–Whitney *U* test was used. A DEG analysis using DESeq2 was conducted with a Wald test, and log2 fold changes were evaluated for statistical significance. A pathway enrichment analysis using the clusterProfiler was performed, with statistical significance analyzed through hypergeometric testing. A TF enrichment analysis using the ChIP-Atlas was analyzed using Fisher’s exact test, comparing observed TF binding site overlaps in DEGs to those in the background genome. Statistical significance was determined and adjusted using the Benjamini–Hochberg method. A *p*-value and adjusted *p*-value threshold of <0.05 was considered statistically significant.

## 5. Conclusions

This study advances our understanding of periodontitis by uncovering key molecular mechanisms and systemic implications through a temporal transcriptomic analysis. We identified critical pathways and transcription factors linking mitochondrial dysfunction, oxidative stress, and immune dysregulation to disease progression and systemic conditions like DM and CVD. These findings highlight potential biomarkers and therapeutic targets, paving the way for precision medicine approaches to address periodontal and systemic health challenges. Future research should validate these discoveries and explore their clinical applications.

## Data Availability

The data have been deposited with links to BioProject accession numbers PRJDB15404 at https://ddbj.nig.ac.jp/search/entry/bioproject/PRJDB15404 (accessed on 5 December 2024).

## Appendix A

**Table A1:** The primer sequencing for qPCR.

Gene	Sequence
*Saa3*	F: AGCAGAGGACTCAAGAGCTG
	R: TAGTTGCTCCTCTTCTCGGG
*Sele*	F: CTGCTGGAGTCATGAATGCC
	R: ACCAGATGTGTGTAGTCCCG
*Col18a1*	F: CCTCTAGGCTGCAGGATCTC
	R: AGGACATCTCTGCCGTCAAA
*Lyz2*	F: TACTGCAGCTCATTCGGTCT
	R: GCTGACTGACAAGGGAGACT
*Cxcl5*	F: TGGGAACTCAAACCTTGGTC
	R: ATCGTTGTTCCCATCCACAT
*Ctsk*	F: CAGCAGAACGGAGGCATTGA
	R: CTTTGCCGTGGCGTTATACATACA
*Tbp*	F: GAGTTGCTTGCTCTGTGCTG
	R: CTGGCTTGTGTGGGAAAGAT
